# Comparative inhibitory profile and distribution of bacterial PARPs, using *Clostridioides difficile* CD160 PARP as a model

**DOI:** 10.1038/s41598-018-26450-0

**Published:** 2018-05-23

**Authors:** Antonio Ginés García-Saura, Rubén Zapata-Pérez, José Francisco Hidalgo, Álvaro Sánchez-Ferrer

**Affiliations:** 1 Department of Biochemistry and Molecular Biology-A, Faculty of Biology, Regional Campus of International Excellence “Campus Mare Nostrum”, University of Murcia, Campus Espinardo, E-30100 Murcia, Spain; 2 Murcia Biomedical Research Institute (IMIB-Arrixaca), 30120 Murcia, Spain

## Abstract

Poly-ADP-ribose polymerases (PARPs) are involved in the regulation of important cellular processes, such as DNA repair, aging and apoptosis, among others. They have been considered as promising therapeutic targets, since human cancer cells carrying BRCA1 and BRCA2 mutations are highly sensitive to human PARP-1 inhibitors. Although extensive work has been carried out with the latter enzyme, little is known on bacterial PARPs, of which only one has been demonstrated to be active. To extend this limited knowledge, we demonstrate that the Gram-positive bacterium *Clostridioides difficile* CD160 PARP is a highly active enzyme with a high production yield. Its phylogenetic analysis also pointed to a singular domain organization in contrast to other clostridiales, which could be due to the long-term divergence of *C. difficile* CD160. Surprisingly, its PARP becomes the first enzyme to be characterized from this strain, which has a genotype never before described based on its sequenced genome. Finally, the inhibition study carried out after a high-throughput *in silico* screening and an *in vitro* testing with hPARP1 and bacterial PARPs identified a different inhibitory profile, a new highly inhibitory compound never before described for hPARP1, and a specificity of bacterial PARPs for a compound that mimics NAD^+^ (EB-47).

## Introduction

Post-translational modifications (TMPs), which are widespread throughout the phylogenetic scale, consist of chemical modifications that occur in proteins catalysed by specific enzymes^1^. TMPs allow cells to produce rapid responses to changes in the environment. Among the different types described in both prokaryotic and eukaryotic cells is the so-called ADP-ribosylation^2,3^, which introduces units of ADP-ribose (ADPr) at the expense of NAD^+^. This reaction is catalysed by a special class of glycosyltransferases, named ADP-ribosyltransferases (ARTs). They were first described in the diphtheria toxin and then in the choleric toxin as a form of interference with important proteins (e.g. elongation factor 2, G proteins, and Rho GTPases), thereby disrupting host cell biosynthetic, regulatory and metabolic pathways as a way of gaining advantage during the infection process^4^. ARTs can be divided into two main groups based on active site amino acids: the so-called ADP-ribosyl transferases cholera toxin-like (ARTCs) and ADP-ribosyl transferases diphtheria toxin-like (ARTDs). The first group includes GPI-anchored extracellular or secreted enzymes containing an R-S-E (Arg-Ser-Glu) motif, which catalyse the mono-ADP-ribosylation (MARylation) of their substrates^5^. The remaining group comprises intracellular ADP-ribosyl transferases able to transfer either a single ADP-ribose residue (H-Y-I/L motif) or several ADP-ribose residues (H-Y-E motif), resulting in linear or branched chains of ADP-ribose (poly-ADP-ribosylation or PARylation)^6^. In the latter group, the invariant Glu (E) is the key catalytic residue that coordinates the transfer of ADP-ribose to the acceptor site, the His (H) forms a hydrogen bond with the N-ribose, and the tyrosine (Y) side chain stacks with the N-ribose and the nicotinamide moiety, thus facilitating the binding of NAD^+^ ^7^. However, when the catalytic glutamate residue is replaced by a small hydrophobic residue in enzymes of the mono-ARTD group (mARTD), a glutamate residue of the substrate is used as the catalytic glutamate, giving rise to a substrate-assisted catalysis to transfer the ADP-ribose moiety. This produces a modified glutamate residue, which is then no longer available for the addition of new ADPr molecules^8^.

PARylation in mammal cells plays a crucial role in cellular functions, including mitosis, DNA repair and cell death^9^. Among the seventeen PARP enzymes identified in the human genome^10^, only Poly(ADP-ribose) polymerase-1 (PARP1 or ARTD1), PARP2, PARP3, PARP4, Tankyrase1 (TNKS1, also known as ARTD5 or PARP5a) and Tankyrase2 (TNKS2, also known as ARTD6 or PARP5b) are capable of catalysing poly-(ADP-ribosyl)ation, whereas PARP10, PARP12, PARP14 and PARP15 are mono-(ADP-ribosyl)transferases^10^. The remaining members of the family, PARP9 and PARP13, appear to be enzymatically inactive^11^. Among them, human PARP-1 (hPARP1) is the most abundant and most active protein in the PARP family, being a nuclear chromatin-associated protein^11^. It is also the best-studied protein in the PARP family since monotherapy with PARP-1 inhibitors selectively kills tumours harbouring deficiencies in *BRCA1* and *BRCA2* genes, which are involved in homologous recombination DNA repair pathway^12^. This ‘synthetic lethality’ has attracted clinical attention over the years as more potent and selective inhibitors have been identified. Several clinical trials are currently being conducted with them as a form of ‘personalized’ cancer therapy^13^.

hPARP1 has a modular architecture comprising six domains^14^. The N-ter site consists of two zinc finger domains (Zn1 and Zn2) that recognize the damaged DNA ends, and a third zinc finger domain (Zn3) that intervenes in DNA-dependent activation^15^. There is also a central BRCA C-terminal-like domain (BRCT) that modulates protein-protein interactions and accomplishes PAR self-modification, and a tryptophan-glycine-arginine (WGR) domain that is important for DNA-dependent activation after interaction with DNA^15^. The last portion of the protein is the catalytic domain, which has an α-helix domain serving in the allosteric regulation (PARP_reg) followed by an ART domain (PARP_cat), which contains the conserved catalytic glutamate^14^.

The last three domains (WGR-PARP_reg-PARP_cat) are also found in hPARP2 and hPARP3 but fused with a variable N-ter tail, as well as in most eukaryotes except for yeasts^7^. Nevertheless, the number of sequences in prokaryotes is reduced to only 28 PARP homologue sequences in 27 bacterial species^16^. Curiously, its activity has only been experimentally tested by western blot with anti-PAR antibodies with a recombinant enzyme cloned from the filamentous predatory gram-negative bacterium *Herpetosiphon aurantiacus*^17^. The above enzyme (HaPARP) was active in the presence of activated DNA and inhibited with the hPARP1 inhibitor KU-0058948^17^. In addition, *H. aurantiacus* also has a DUF2263 protein (UniProt code: T3D766) that is capable of effectively removing PAR^17^, and whose sequence contains a poly (ADP-Ribose) glycohydrolase (PARG) signature (GGG-X_6–8_QEE)^18^. Thus, the presence of both enzymes in the same microorganism suggests that certain bacteria may have a functional PAR metabolism^16^. Another example of a microorganism with both putative PARP and PARG homologues is the rod-shaped, Gram-positive spore-forming anaerobic bacillus *Clostridium difficile* CD160, now reclassified as *Clostridioides difficile* CD160^19,20^.

*C. difficile* is a nosocomial opportunistic antibiotic-associated pathogen of humans responsible for a spectrum of diseases known collectively as *C. difficile* infections (CDI), which range from a mild self-limiting diarrhoea to pseudomembranous colitis and toxic megacolon^21^. These pathologies often result in death, causing 29,000 deaths and costs in excess of US 6.0 billion dollars per annum in the USA alone^22^. The major virulence factors of this microorganism are the potent A-type monoglycosyltransferases toxins A (TcdA) and B (TcdB) that attach glucose to Rho proteins using UDP-glucose as a cosubstrate^23^, and the *C. difficile* transferase (CDT) toxin, frequently produced by so-called hypervirulent strains. CDT is a two component toxin, CDTa being involved in the ADP-ribosylating activity and the CDTb in binding^21^. All the above-mentioned toxins are located within two defined loci, the PaLoc (Pathogenic Locus, with *tdcA*, *tdcB*, *tdcR*, *tdcE* and *tdcC* genes) and the CdtLoc (with *cdtA* and *cdtB* genes)^24^. Interestingly, non-toxigenic *C. difficile* strains (which lack the genes for toxins A and B and the binary toxin) are also relatively common, although little is known about their biology^25^. The same is the case with *C. difficile* CD160, which was isolated from a clinical stool at the University of Michigan Medical Centre, to test for its toxigenicity. However, the evaluation of this isolate by PCR with two different primers revealed that it was negative for *C. difficile* toxin genes A and B (*tcdA*^−^ and *tcdB*^−^). Nevertheless, *C. difficile* CD160 was selected for sequencing because CE-PCR analysis showed it to be a unique ribotype (Prof. Seth Walk, Montana State University, USA, personal communication). Apart from this uniqueness, only one protein has been studied from this microorganism, a type IV pilin region (PilA1) with an unusual conformation compared with PilA1 from *C. difficile* R20291 and NAP08 strains^26^.

The present work makes a comprehensive phylogenetic analysis of bacterial PARPs to better understand the domain organization of these enzymes. Surprisingly, the domain organization in *C. difficile* CD160 PARP (CdPARP) differed from that of other clostridial PARPs. A detailed study of this anomalous distribution suggested the fact that *C. difficile* CD160 long ago diverged from other *C. difficile* strains and also defined a new toxigenotype. In addition, the presence of an active DUF2263 protein in *C. difficile* CD160 (CdPARG) revealed the existence of a functional PAR metabolism. Kinetic characterization also revealed CdPARP to be a highly active enzyme, with an activity that was 3-fold higher than that observed previously in HaPARP. Finally, the inhibition profile of the bacterial PARPs studied was also different from that of hPARP1, providing new information for the development of novel bacterial inhibitors with well-defined selectivity.

## Results

### CdPARP shows an atypical PARP domain organization compared with other clostridial PARPs

**Figure 1:**
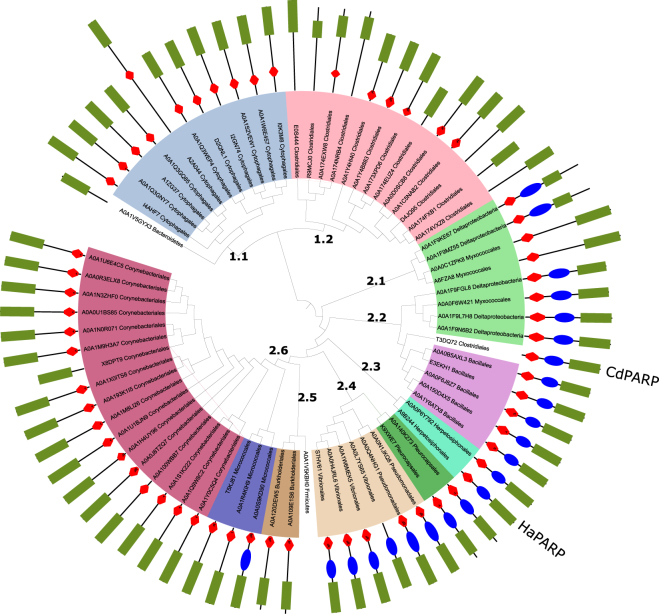
Phylogenetic analysis of bacterial PARPs. The Neighbour-Joining (NJ) tree was obtained from 1000 replicates. Protein domain architecture is shown behind each protein code: WGR domain (Red), PARP regulatory domain (Blue), and PARP catalytic domain (Green). Protein codes are summarized in Supplementary Table S1.

A phylogenetic analysis of bacterial PARPs was carried out to compare the sequence and domains of CdPARP, using the data available in the UniProt and NCBI databases. Seventy-two sequences were found containing at least the PARP domain (PARP_cat) (Supplementary Table S1) compared to the 28 previously described in the bibliography^16^. These sequences are distributed in six phyla (Actinobacteria, Bacteriodetes, Choloflexi, Cyanobacteria, Firmicutes, and Proteobacteria) and twelve orders (Fig. 1). The phylogenetic tree shows that these sequences are divided into two large clades. Clade 1 groups all the sequences of two orders, Cytophagales (Clade 1.1) and Clostridiales (Clade 1.2), with the exception for the sequence of CdPARP, which is in Clade 2 (see below). Almost all members of Clade 1 are characterized by the presence of WGR and PARP_cat domains without a defined PARP_reg domain. However, when these sequences are modelled, their corresponding three-dimensional structures show the presence of a helical domain, which could fulfil the same regulatory role as the canonical PARP_reg domain does. Clade 1.1 also contained the longest bacterial PARP used in this study (764 amino acids), which corresponded to one of the two PARPs reported for the bacterium *Microscilla maritima*, and whose origin seems to be linked to a fusion of a tail of 350 residues in the N-ter. In addition, *Bacteroidetes bacterium* ADurb.BinA174 PARP (UniProt code: A0A1V5GYX3) is a sister clade of Clade 1.1. On the other hand, Clade 2 is characterized by the balanced presence of both WGR-PARP_reg-PARP_cat and WGR-PARP_cat domain configurations. In the latter case, modelling of the sequences again shows the presence of a helical domain such as that found in the proteins of Clade 1. An example of this dichotomy can be found in Clade 2.1, which consists of four Deltaproteobacteria sequences with the two domain organizations described above. The rest of the Deltaproteobacteria found in the databases were encountered in Clade 2.2, where they formed a sister group with different bacilli. All members of this Clade 2.2, including CdPARP, have the canonical WGR-PARP_reg-PARP_cat organization, an architecture also found in Clades 2.3 and 2.4, formed by proteins belonging to the orders Herpetosiphonales (Clade 2.3), Pleurocapsales, Pseudomonadales and Vibronales (Clade 2.4). By contrast, in Clade 2.5, constituted by two Burkholderiales and one Firmicutes bacterium, the predominant organization was the WGR-PARP_cat. Finally, Clade 2.6 contains all the actinobacterial sequences found in this study, divided into two large groups, belonging to the Micrococcales and Corynebacteriales orders, the latter being the most abundant. The only member of this group with the three domains is the PARP of *Leifsonia* sp. Leaf264, a microorganism of the *Arabidopsis* leaf microbiota.

**Figure 2:**
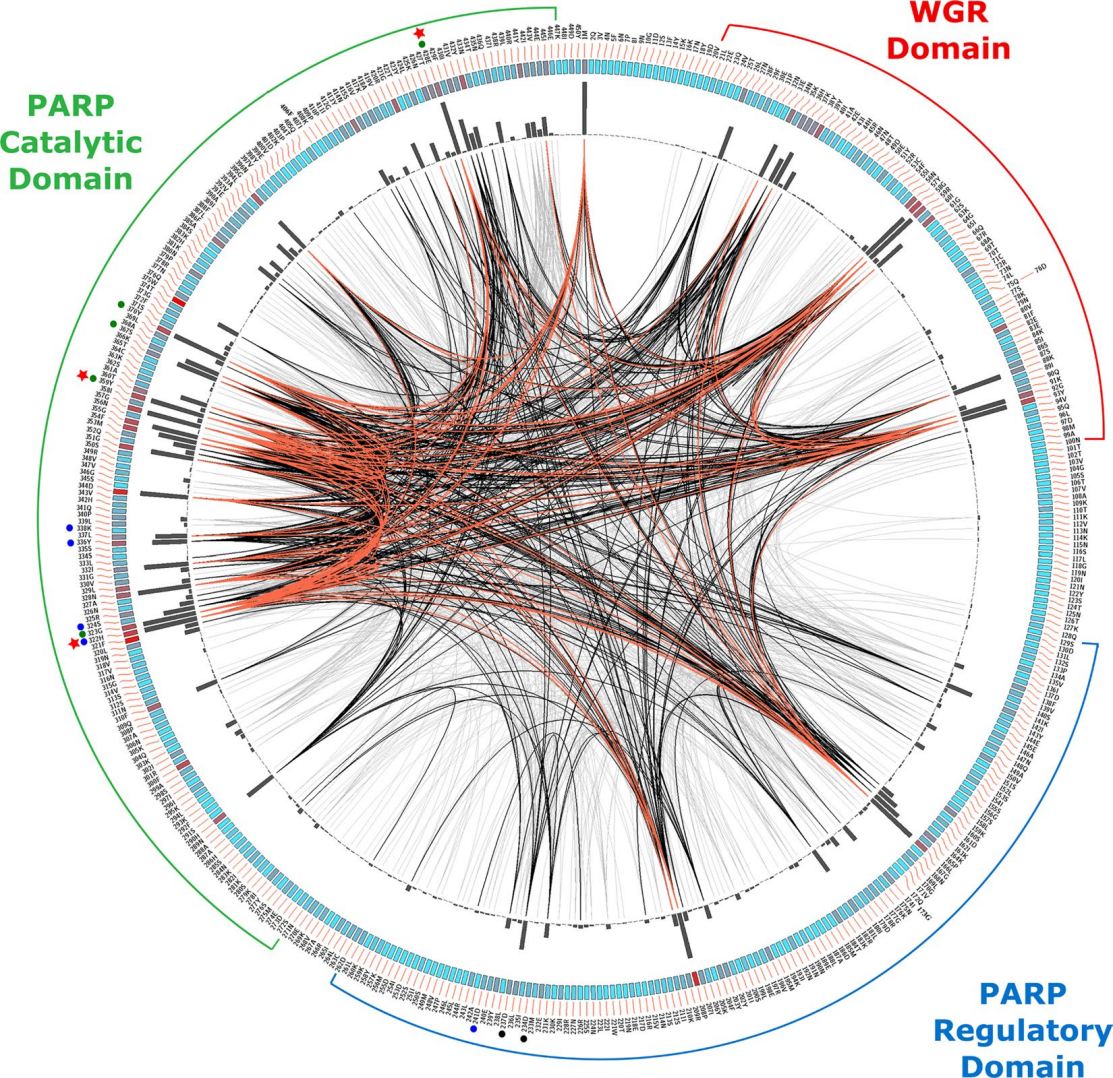
Circos representation of the bacterial PARPs. The outer ring shows the amino acid code corresponding to CdPARP (Uniprot code; T3DQ72). Coloured rectangular boxes of the second circle indicate the KL (Kullback-Leibler) conservation score (from red to cyan, red: highest; cyan: lowest)^27^. The third circle shows the cMI (cumulative Mutual Information score) scores as histograms. Lines in the centre of the circle connect pairs of positions with MI (Mutational Information) score > 6.5. Red lines represent the top 5%, the black lines between 70 and 95%, and the grey lines account for the last 70%. Sequence distribution of WGR, PARP regulatory and PARP catalytic domains are shown. Amino acids belonging to the catalytic triad are marked with red stars, the amino acids from the NI site, PH site and AD site are marked with green, black, and blue circles, respectively.

The MISTIC server^27^ (mutual information server to infer coevolution) was used to analyse and visualize the extent of the coevolutionary relationship between two positions in the bacterial PARP protein family by using the information contained within the above MSA (multiple sequence alignment)^28^. The mutual information (MI) obtained is usually used to find structurally or functionally important positions in a given protein fold family^29,30^. The residue-based Kullback-Leibler conservation score (Fig. 2, coloured rectangles) and cumulative mutual information score (cMI) for each residue (Fig. 2, black histograms) were fairly similar, both revealing that highly coevolving residues were primarily localized in the donor site of the catalytic domain (N316-Y432 in CdPARP; Fig. 2), and in the DNA binding site of the WGR domain (V20-N100, Fig. 2). In addition, some residues of PARP_reg domain are also highly coevolving (K163-Y239, Fig. 2). The three main regions of the donor site corresponding to a nicotinamide (NI site; G323, Y359, S367, Y370 and E428; Fig. 2, green circles), a phosphate (PH site; D234 and D237; Fig. 2, black circles) and an adenine-ribose binding (AD site; D241, H322, S324, Y336 and K338; Fig. 2, blue circles) sites are in zones characterized by high cMI scores (Fig. 2). Among these residues, especially conserved are those involved in the catalytic triad (H322, Y359 and E428; Fig. 2, red stars). The circos representation also shows three areas of high cMI scores in the WGR domain (N34-Y39; Y57-G61 and K88-Y93), which are related with the DNA-dependent activity of PARPs, the highest being those corresponding to G58 and R59. Of note is that, in the bacterial sequences used, the first position of WGR zone is mostly occupied by a tyrosine (Y), followed by a tryptophan (W) or a phenylalanine (F). In the PARP_reg domain, two leucines (L181 and L236) involved in the stabilization of PARP_reg hydrophobic core are also highly conserved. These two leucines correspond to L269/L233 and L321/L286 in hPARP2/hPARP3, respectively, whose alanine mutants showed an increase in DNA-independent activity, mimicking of distortion of the helical domain produced after DNA binding^31^.

The results described above, along with those found in Supplementary Fig. S1, which shows that the 12 bacterial sequences corresponding to each of the 12 different orders found are grouped together with DNA-repairing eukaryotic PARPs in Clade 1^16,32^, may indicate that the proposed organization into three structural domains for the last common ancestor of all eukaryotes may occasionally have been acquired by bacteria through horizontal gene transfer, in some cases remaining intact and in others changing their canonical sequence but not theirs helical structure. This conclusion corroborates what Ahel’s group previously proposed^16^.

### Genetic organization of *C. difficile* CD160 reveals a new toxinotype

**Figure 3:**
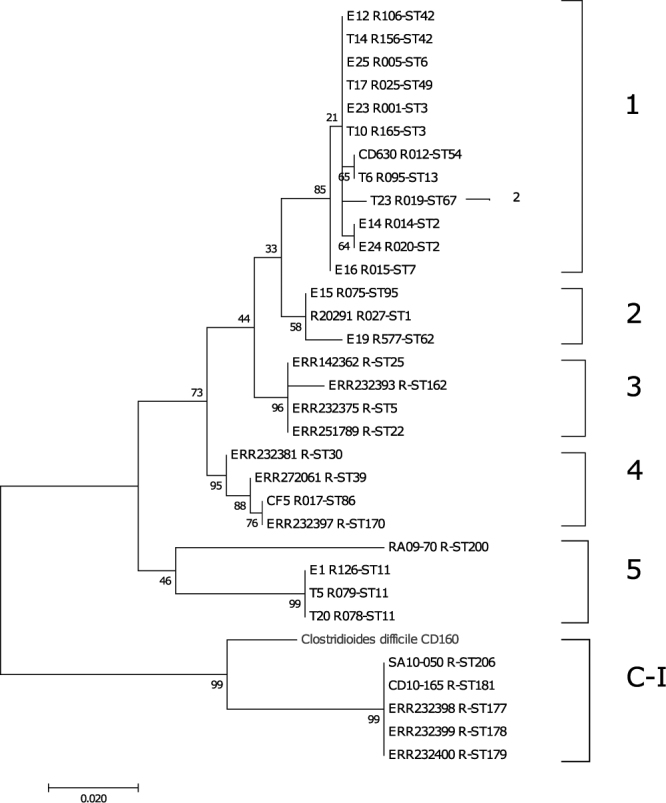
Phylogenetic analysis of the *C. difficile cdu1* genes. Maximum likelihood tree with 1000 replicates was constructed using representative clade strains^24^.

In order to explain the above anomalous localization of CdPARP in a clade other than that of Clostridiales, a maximum likehood tree from the *cdu1* genes of at least three *C. difficile* strains for each of the six clade already described^24^ was generated, including for the first time the corresponding gene from *C. difficile* CD160 (Uniprot code: QEW_0946) (Fig. 3). The result showed that *C. difficile* CD160 forms a sister group within Clade C-I, a highly divergent lineage containing toxigenic and non-toxigenic strains^24,33^, including SA10-0505 and CD10-165 strains^24^. These latter strains, along with toxinotypes XXX and XXXI, are a group of *C. difficile* that lack a complete *tcdA* gene while preserving *tcdB* and CDT genes (toxinotype A^−^B^+^CDT^+^); however, CD160 strain is a completely new toxinotype (A^−^B^+^CDT^−^) with the same PaLoc organization as toxigenic SA10-0505 and CD10-165 strains (*cdtR*, *tcdR*, *tcdB*, *tcdE*) but without *cdtB*-*cdtA* genes (Supplementary Fig. S2). CD160 strain also contains the endolysin *cwlH* gene corresponding to a N-acetylmuramoyl-L-alanine amidase, which corroborates the phage origin of the PaLoc^24^. In addition, the homology of the TcdR (UniProt code: T3DH32) and CdtR (UniProt code: T3DFV6) regulators in CD160 and those of the reference strain CD630 (Uniprot codes: Q189K4 and Q182U3 respectively) is low (73% and 52%, respectively), but in the range of values described for SA10-0505 and CD10-165 strains (70% and 62%, respectively)^24^. These low values suggest a long-term divergence of CD160 strain compared not only with those of the *C. difficile* strains already studied but perhaps also with other clostridiales; indeed, a different origin for the *C. difficile* CD160 PARP gene is plausible.

### *C. difficile* CD160 has functional PAR metabolism

In order to test whether or not CdPARP is a *bona fide* DNA-dependent PARP as predicted by *in silico* analysis, it was cloned into pET28a vector and transformed into *E*. *coli* Rosetta 2(DE3). In addition, due to the great variability that the bibliography mentions for kinetic parameters depending on the assay used, two additional PARPs with demonstrated DNA-dependent PARP activity were also cloned: the full-length hPARP1 (1–1014) into pET24b vector and the bacterial HaPARP into pET28a vector^17,34^. The soluble recombinant proteins obtained at 20 °C after induction with IPTG were isolated in three simple steps, as described in Materials and Methods. SDS-PAGE pure enzymes were obtained with their corresponding molecular masses of 113.5 kDa, 52 kDa and 47 kDa for hPARPl, CdPARP and HaPARP, respectively (Supplementary Fig. S3). CdPARP rendered the highest yield of purified protein (4.5 mg/L culture), followed by HaPARP (3.9 mg/L culture), whereas hPARPl had the lowest (1.2 mg/L).

**Figure 4:**
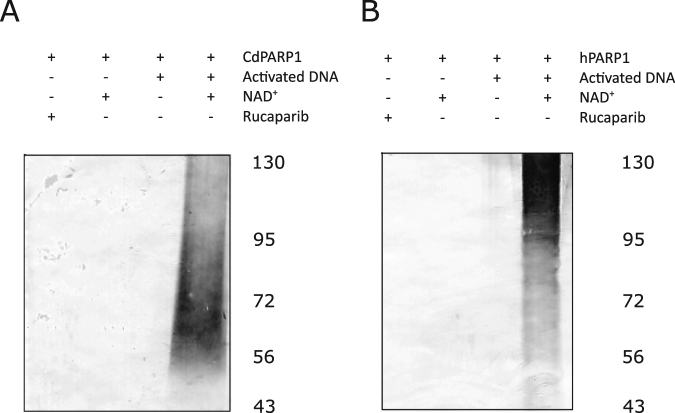
PARylation assay of CdPARP and hPARP1. It was carried out with CdPARP (**A**) and hPARP1 (**B**) as control in the presence of absence of NAD^+^, activated DNA and the PARP inhibitor rucaparib. Numbers on the right margin indicate protein markers (kDa).

HaPARP and hPARP1 DNA-dependent automodification has previously been demonstrated by western blot and PAR antibodies^17^. When this process was assayed with CdPARP, using hPARP1 as a control, both PARPs showed a noticeable shift in mobility as seen by western blot using both NAD^+^ and activated DNA as substrates (Fig. 4), thus indicating poly(ADP-ribosyl)ation of the corresponding PARP. This process was also sensitive to rucaparib, a well-known hPARP1 inhibitor (Fig. 4). In addition, the PARylation shown by hPARP1 and CdPARP was abolished by recombinant protein corresponding to the *Clostridioides difficile* CD160 QEW_4455 gene (Uniprot code: T3D766). This uncharacterized protein has a DUF2263 domain similar to that of hPARG and HaPARG, which also reverses PARylation (Supplementary Fig. S4). These results demonstrate that *C. difficile* CD160 has a functional PAR metabolism with both functional CdPARP and PdPARG, as has also been described for *H. aurantiacus*^17^.

### Biochemical characterization of recombinant CdPARP

The kinetic parameters of hPARP1 were determined using different assay methods with PARP alone or combined with histones, together with NAD^+^ (radioactive, biotinylated or as ADP-ribose-pNP) and with activated DNA or double-stranded DNA oligomers^35,36^. This resulted in different K_M_ (0.06–0.27 mM), *k*_cat_ (0.41 s^−1^) and *k*_cat_/K_M_ (1.47–6.95 mM^−1^ s^−1^) values^37^. Since no kinetic parameters have been described for any bacterial PARP, recombinant hPARP1 was used as a reference to validate the fluorescent method used in this work, which measures the consumption of substrate by the chemical conversion of the remaining NAD^+^ into a stable fluorescent condensation product upon treatment by acetophenone in ethanol at basic pH, followed by heating at acidic conditions^38,39^. Among all the enzymes used, hPARP1 was the most active with a *k*_cat_/K_M_ at 3.06 × 10 mM^−1^ s^−1^, value 5.2- and 17-fold higher than those of CdPARP and HaPARP, respectively (Table 1). These results highlighted the important contribution of the three zinc fingers and BRCT domain for the activity, which results in a lower K_M_ and a higher *k*_cat_ (Table 1). As regards bacterial PARPs, both showed a similar K_M_ but the *k*_cat_ was higher in CdPARP, resulting in a 3-fold higher catalytic efficiency compared with HaPARP (Table 1). These data, together with the purification yield of CdPARP, point to this enzyme as a promising biocatalyst for PAR production.

**Table 1:** Kinetic parameters of hPARP1, CdPARP and HaPARP. Literature values are from Altmeyer *et al*.^37^.

	K_M_ (mM)	*k*_cat_ (s^−1^)	*k*_cat_/K_M_ (mM^−1^ s^−1^)
hPARP1 literature	0.06–0.28	0, 41	1.47–6.95
hPARP1	0.20 ± 0.04	0.60 ± 0.02	3.06
CdPARP	0.37 ± 0.02	0.21 ± 0.01	0.58
HaPARP	0.45 ± 0.09	0.08 ± 0.02	0.18

The thermal stability of the above PARPs was also studied under different conditions by monitoring Sypro Orange fluorescence while heating the samples, and determining the midpoints of the transitions (melting temperature or *T*m)^30^. Previous investigations showed that this assay provides a good estimate of binding affinity, optimal storage pH and the suitable protein stabilizers to be used^40,41^. To carry out such study, the melting temperature of each enzyme in MilliQ^®^ water was taken as a reference, and thus the increment in *T*m (Δ*T*m) was calculated. All enzymes showed a similar *T*m in MilliQ^®^ water (40–45 °C) (Supplementary Table S2), hPARP1 being the most stable. When the effect of pH on enzyme stability was studied (Supplementary Table S2), both hPARP1 and CdPARP showed higher increments in *T*m at pH 7.5, while, in the case of HaPARP the values were higher at pH 8.0–8.5. However, pH values below pH 7.0 or above pH 8.5 decreased the stability of these enzymes. The protein-stabilizing compounds used (ammonium sulphate and hydroxyectoine), while producing an increase in their *T*m with all the enzyme, presented a differential effect between them (Supplementary Table S2). Thus, ammonium sulphate was the best stabilizer for CdPARP, while hydroxyectoine was the best stabilizer for HaPARP and hPARP1. This information may be relevant for the case of hPARP1 as it is a commercial enzyme. In fact, when comparing the stability of hPARP1 in the presence of glycerol (10%) and hydroxyectoine (200 mM) at 4 °C, the latter compound preserved 100% of the activity after 24 h, while in the presence of glycerol it is reduced to 75% (Supplementary Fig. S5). This proportion is maintained even at 72 hours, where the activity in the presence of glycerol is only 8% compared with 32% in the presence of hydroxyectoine (Supplementary Fig. S5). Finally, NAD^+^ and ADP-ribose had a destabilizing effect at 1 mM, but nicotinamide and the inhibitor 3-aminobenzamide at the same concentration increased the *T*m of all enzymes, especially in the case of HaPARP, where the *T*m increase was 7 °C (Supplementary Table S2). This increase in *T*m due to the presence of 3-aminobenzamide has previously been observed with an hPARP1 construct comprising the domains PARP_reg-PARP_cat (K654-L1013), but not with the entire protein^40^.

### Bacterial PARPs are specifically inhibited by a compound that mimics NAD^+^

The inhibitory profile of bacterial PARPs was assessed using a subset of small molecules that were originally selected following an *in silico* high-performance screening campaign on two complementary libraries of 50,000 compounds each (DIVERSet-EXP and DIVERSet-CL, ChemBridge), designed to provide the greatest coverage of pharmacophore while maintaining structural diversity. In addition, nine well-known hPARP1 inhibitors were also tested using a more sensitive fluorescent method due to the high potency of some of them. Thus, the remaining NAD^+^ was amplified by coupling the enzymes alcohol dehydrogenase and diaphorase. Each time the NAD^+^ was recycled through these enzymes, a highly fluorescent resorufin molecule was generated. This assay was first used to determine the enzyme activity of NMN-adenyltransferase and ADP-ribosyl cyclase^42,43^.

The selected PARP inhibitors and analogues were initially screened at 10 µM and 50 µM with both hPARP1 and bacterial PARPs (CdPARP and HaPARP), respectively (Supplementary Table S3). Of the 44 compounds analysed, twenty-three showed more than 50% inhibition with hPARP1 (Supplementary Table S3). Half-inhibitory concentration (IC_50_) values were calculated for the first 17 hPARP1 inhibitors, obtaining very similar values for the 8 inhibitors already described in the bibliography (Table 2). After the 5 highly selective known inhibitors (rucaparib, ABT-888, PJ-34, EB-47 and DPQ) surprisingly, four compounds derived from 4-substituted 3-nitrophenyl-1(2 H)-phthalazinone appeared (Table 2; Supplementary Fig. S6). Among them, standing out for its selectivity, was compound **7655698** with an IC_50_ of 81 nM, which, to the best of our knowledge, has never been used as hPARP1 inhibitor, and which could be optimized to increase its potency and selectivity. The others members of this family of compounds with IC_50_ values between 136–481 nM have been tested as modulators of cytokine activity^44^ or maternal embryonic leucine zipper kinase^45^, and only one (**7660328**) has been used to calculate its *T*m increment with a hPARP1 construct (K654-L1013)^46^. This was also the case with compound **7650155** (1,3-dioxo-2,3-dihydro-1H-benzo[de]isoquinoline-5-sulfonamide)^46^, which together with another two N-alkyl derivatives (**7670490** and **9019116**) that had never before been tested with hPARP1, belong to a second family of compounds with IC_50_ values between 403–676 nM (Table 2; Supplementary Fig. S6).

**Table 2:** IC_50_ values of well-known and new compounds against hPARP1, PdPARP and HaPARP.

Compound	hPARP1		CdPARP	HaPARP
	Literature IC50 (nM)	IC50 (nM)	IC50 (µM)	IC50 (µM)
Rucaparib	1.4^56^	12.1 ± 1.7	5.3 ± 0.7	7.2 ± 0.7
ABT-888	5.2^57^	21.0 ± 1.9	>50	>50
PJ-34	20^58^	34.5 ± 3.1	32.4 ± 9.8	50.4 ± 6.4
EB-47	45^47^	37.2 ± 4.9	0.86 ± 0.07	1.0 ± 0.1
DPQ	40^59^	73.8 ± 8.2	25.3 ± 7.1	>50
7655698	—	81.2 ± 21.3	—	—
7650649	—	136.1 ± 41.1	—	—
7651361	—	168.2 ± 44.1	—	—
7642078	—	181.9 ± 21.4	—	—
4-amino 1.8-naphthalimide	180^60^	247.6 ± 35.9	—	—
7669941	—	308.4 ± 21.3	—	—
7670490	—	403.7 ± 53.4	—	—
7650155	—	458.9 ± 74.7	—	—
7660328	—	481.3 ± 30.1	—	—
TIQ-A	450^61^	596.3 ± 31.4	15.7 ± 4.1	7.9 ± 1.7
9019116	—	676.8 ± 32.7	—	—
XAV-939	2200^62^	726.2 ± 156.1	>50	>50

Literature values are from different authors^47,56–62^.

**Figure 5:**
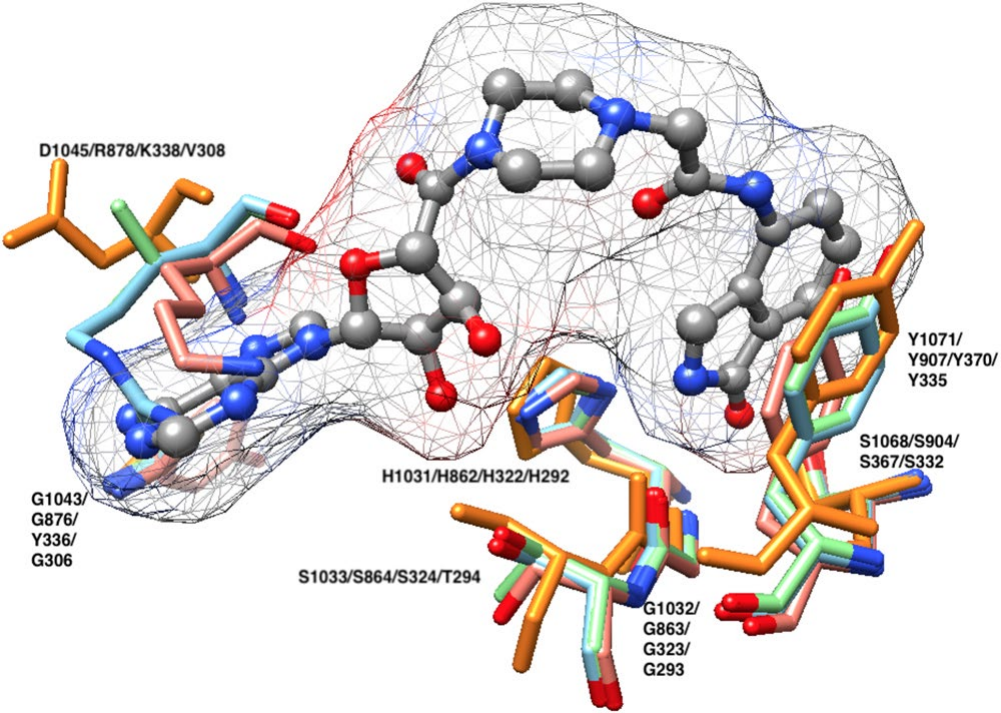
PARP inhibitor EB-47 binding site. Structural alignment of TNKS2 (PDB: 4BJ9, orange), hPARP1 (PDB: 4OPX, cyan) and modelled CdPARP (salmon) and HaPARP (green) was carried out using chimera^54^. hPARP1 AD site is represented by R878, G876, H862 and S864, whereas Ni site by G863, S904 and Y907, respectively.

As regards bacterial PARPs, only five compounds showed inhibition in excess of 50% with CdPARP, and three compounds with HsPARP (Supplementary Table S3). However, only rucaparib and EB-47 were able to inhibit them completely. When IC_50_ values were determined, rucaparib showed values of 5.3 and 7.2 µM, while EB-47 showed values of 0.8 and 1.0 µM, respectively (Table 2). The latter inhibitor was designed to mimic the NAD^+^ within the hPARP1 substrate-binding site^47^ and has been co-crystallized with tankyrase-2 with an IC_50_ of 45 nM^48^. When a structural alignment of this tankyrase-2 with EB-47 (PDB: 4BJ9) was carried out, both with hPARP1 (PDB: 4opx) and with the modelled CdPARP and HsPARP, it was observed that at the nicotinamide site the residues that form the hydrogen bonds (G1032 and S1068; TKNS2 numbering) and produce π-π stacking (Y1071) with isoindolinone moiety are also conserved in the rest of the studied PARPs (Fig. 5). In addition, the residues involved in binding the ribose hydroxyls (H1031 and S1033) and the two H-bonds with the adenosine (G1043 and D1045) were also structurally conserved (Fig. 5). The different IC_50_ of the above PARPs and TKNS2 could be related with the specific interaction of EB-47 with the D-loop of each enzyme, as previously described for TKNS2^48^.

## Discussion

Poly-ADP-rybosilation is a remarkably, well-conserved post-transcriptional modification in eukaryotes, but it is less common in bacteria. This was also observed in the phylogenetic studies carried out, mainly focusing on the evolutionary distribution of the poly(ADP-ribose) polymerases in eukaryotes. In this study, we looked into bacterial PARPs, not only as regard their phylogenetic distribution, by representing their first phylogenetic three (Fig. 1), but also the key amino acids responsible for catalysis, and DNA-dependent activation, using the coevolutionary relationship between two positions provided by the MISTIC server. With this aim, we focused on the *C. difficile* CD160 putative protein corresponding to QWE_4625 gene, since its protein in the PFAM database indicates the presence of a WGR-PARP_reg-PARP_cat canonical organization. The updated phylogenetic analysis was carried out with 72 sequences rather than the 27 previously used. Unexpectedly, the result showed that even when PFAM did not found in most of the protein sequences the PARP_reg domain, the corresponding modelled sequences displayed a similar helical domain to the canonical PARP_reg domain, which is necessary for the destabilization of PARP_cat domain after DNA-binding at the WGR domain. The study of this last domain produced two important findings: an YGR domain exits in most of bacterial PARPs along with other highly coevolved amino acid, which have never before been studied, such as G92 and Y93 (Fig. 2). In addition, the organization of the CdPARP anomalous domain compared with that of other clostridial PARPs suggests a long-term divergence of CD160 strain, based on its *cdu*1 gene phylogeny (Fig. 3) and homology in TcdR and CdtR. Of note too, is that the latter, poorly studied strain (just one paper mentions it), presents a new toxinotype never before described (A^−^B^+^CDT^−^) with an atypical “Bi-Toxin Paloc” comprising only the *CdtR*, *tcdR*, *tcdB*, *tcdE*, *CwlH* genes. Surprisingly, this PaLoc organization differs from that of the closely related SA10-50 and CD10-165 strains^24^. Furthermore, this microorganism has also a functional *bona fide* PAR metabolism, not only by the existence of PARP (Fig. 4) and PARG (Supplementary Fig. S5), but also by the presence of a putative MacroD (T3DIR9), which is the enzyme predictably responsible for the removal of the terminal ADP-ribose unit after PARG has acted, giving rise to a completely unmodified protein^49^. Surprisingly, no ARH3-like protein was found in UniProt for *C. difficile* CD160, whereas three genes were found in *H. auranticus* ATCC 23779 (Haur_3413, Haur_2362 and Haur_0057), in addition to the existing three MacroD genes (Haur_0008, Haur_4555 and Haur_2355). The presence of different genes involved in the PAR metabolism in Bacteria, together with the presence of sirtuins-like genes would seem to be rather more than a random horizontal gene transfer phenomenon, and more likely an adaptive advantage that has been preserved over time. Such an advantage has been recently described in a new toxin-antitoxin (TA) system, which possibly contributes to bacterial persistence^50^. The latter system is based on the combined action of a toxin protein (DarT) that specifically leads to a sequence-specific ADP-ribosylation on single-stranded DNA and an antitoxin macrodomain protein (DarG) that reverse such DNA modification^50^.

Finally, our data revealed different inhibitory profiles between hPARP1 and bacterial PARPs, the latter enzymes being specifically inhibited by EB-47, a compound that mimics NAD^+^ (Table 2). However, the major discovery in this study was a compound, never before used with PARP, that selectively inhibited hPARP1 (compound 7655698) but not the bacterial PARPs. The absence of Pan-PARP inhibition for bacterial PARPs, as produced by rucaparib (Table 2), makes this compound as an ideal selective inhibitor for seeking further improvements in its potency. Taken together, the above described results will provide the foundation for new future bacterial PAR metabolism studies and the development of new specific PARP inhibitors.

## Materials and Methods

### Protein expression and purification

Bacterial and human PARP and PARG proteins were expressed from the pET28a vector (EMD Millipore, Madrid, Spain) bearing an N-terminal His-tag, with the exception of the hPARP1 (1–1014), which has a C-terminal His-tag and was kindly provided by J. M. Pascal (Université de Montréal, Montréal, QC) in a pET24b vector. PARP (QEW_4625) and PARG (QEW_4455) genes from Clostridioides difficile CD160 (BioProject and SRA Accession numbers PRJNA85757 and SRR593185, respectively) were obtained from GenScript (Piscataway, USA). Genomic DNA from Herpetosiphon aurantiacus DSM785, purchased from DSMZ (Braunschweig, Germany), was used to clone PARG (Haur_1618) and PARP (Haur_4763) genes. hPARG-pCMV6-XL5 construct was purchased from Origene (Rockville, USA). Genes were amplified by PCR using KAPA HiFi DNA polymerase and the corresponding primers, including *Nhe*I and *Xho*I restriction site extensions. The cloning and transformation techniques used were essentially those previously described^51^.

All PARP constructs were expressed in Escherichia coli strain BL21(DE3) Rosetta2. The clones containing the constructions were grown in 1 litre of Terrific Broth (TB) medium supplemented with kanamycin (50 µg/mL) and chloramphenicol (30 µg/mL). When the culture reached an optical density of 2.5 at 600 nm, it was induced with 0.2 mM isopropyl-β-D-thiogalactoside (IPTG) for hPARP1 or with 0.1 mM IPTG for both HaPARP and CdPARP for 16 h at 20 °C with constant shaking. Pelleted cells were resuspended in lysis buffer (50 mM HEPES pH 7.5, 250 mM NaCl, 1 mM β-mercaptoethanol, 1 mM PMSF, 1 mM benzamidine and cOmplete EDTA-free protease inhibitor cocktail) before being disrupted by sonication (450-D Sonifier, Branson). After ultracentrifugation (40000 *g*, 40 min), the supernatant was incubated with Ni-NTA beads (Macherey-Nagel, Germany) at 4 °C for 1 h. Then, they were washed with lysis buffer, and the protein was eluted with the same buffer containing 250 mM imidazole. The PARP-containing fractions were loaded onto a HP heparin column coupled to a FPLC chromatography system (ÄKTA Prime Plus, GE Lifesciences)^52^, and eluted with 50 mM Tris-HCl pH 7.5, 1 M NaCl, 1 mM EDTA and 1 mM β-mercaptoethanol. Finally, the protein was loaded onto a Superdex 200 HiLoad 16/600 column (GE Lifesciences), obtaining an electrophoretically pure enzyme. PARP aliquots were stored at −20 °C with 10% glycerol.

All PARG constructs were expressed as above described but induced with 0.2 mM IPTG when the culture reached an optical density of 4.0 at 600 nm in TB medium. After ultracentrifugation (40000 *g*, 40 min), the resulting supernatant was purified in two steps comprising a Ni^2+^-chelating affinity chromatography (HiPrep IMAC 16/10 FF column) followed by gel filtration onto a Superdex 200 HiLoad 16/600 column (GE Life Sciences). PARG aliquots were also stored at −20 °C with 10% glycerol. The protein concentration was determined using Bradford reagent (Bio-Rad) and BSA as standard.

### PARP activity assays

PARP activity was determined by a fluorescence-based assay based on the reaction of N-alkylpyridinium compounds, such as NAD^+^, with acetophenone to give rise a fluorescent compound after heating in acid^38^. The hPARP1 automodification reaction was allowed to proceed in a black 96-well fluorescence plate (Greiner Bio-One, USA) for 20 min at 25 °C in a reaction medium containing 90 nM of hPARP1, 1 mM NAD^+^ (Trevigen), 75 µg/mL of activated DNA (Trevigen), and PARP assay buffer (100 mM Tris-HCl pH 8.0, 10 mM MgCl and 1 mM DTT) in a final volume of 60 µL. The NAD^+^ present after the reaction was then determined by the addition of 20 µL of an aqueous 2 M KOH solution and 20 µL of a 20% acetophenone solution in ethanol. The plate was incubated at 4 °C for 10 min. Then, 80 µL of formic acid 88% was added, incubated at 110 °C for 5 min and allowed to cool for 30 min. Fluorescence was measured on Synergy HT equipment (Biotek Instruments) at an excitation wavelength of 360 nm and emission wavelength of 445 nm. HaPARP and CdPARP activity assays were carried out as above but at 30 °C for 1 h and at enzyme concentrations of 850 nM and 500 nM, respectively. K_M_ values were estimated using plots of initial rates *vs*. NAD^+^ concentrations.

Due to the potency of some of them, the inhibitors were tested using a more sensitive cycling assay involving alcohol dehydrogenase (ADH) and diaphorase (DP)^42,43^. ADH reduces the NAD^+^ to NADH and oxidizes ethanol to acetaldehyde, while DP turns NADH back into NAD^+^ with the reduction of resazurin, providing resorufin, which has an excitation maximum at 544 nm and an emission maximum at 590 nm. The reaction mixture contains enzyme (90 nM hPARP1 or 0.5 µM CdPARP or 0.85 µM HsPARP), 100 nM NAD and 75 µg/mL of activated DNA in PARP assay buffer. The inhibitors were added at different concentrations at a constant volume of 0.3 µL of DMSO. The PARP automodification reaction was allowed to proceed for 20 min at 25 °C (hPARP1) or 60 min at 30 °C (CdPARP and HsPARP). Then, 30 µL of cycling reagent, containing 2% ethanol, 50 µg/mL ADH, 50 µM Resazurin, 5 µg/mL DP, 10 µM FMN in PARP assay buffer was added. The enzyme-coupled reaction was measured over 15 min in a Synergy HT (Biotek Instruments). The compounds were screened at 10 μM (hPARP1) or at 50 μM (CdPARP and HsPARP) in triplicate, and the best compounds were selected for measuring their corresponding IC_50_ values, which were estimated using dose-response curve fitting. The reported values represent means ± SE of the fits of the curves based on triplicate experiments, each determined on the basis of three replicates.

### Western blot

PARP automodification was also assayed by western blot in a reaction containing different concentrations of enzyme (250 nM HaPARP, 200 nM CdPARP, or 85 nM hPARPl) in assay buffer, with or without NAD^+^ (1 mM), activated DNA or rucaparib (1 µM), respectively. The reactions were allowed to proceed for 1 h at 25 °C. PAR-mediated PARG hydrolysis was analysed using the above-mentioned PARP automodification reaction after stopping it with rucaparib, followed by the addition of PARG enzymes (500 nM) and incubation for 1 hour at 30 °C. The above reactions were then run on 7–10% SDS-PAGE gels and transferred to a nitrocellulose membrane. The presence of poly ADP-ribose (PAR) was detected by the use of rabbit polyclonal anti-PAR antibodies (1:1000; Trevigen), and goat anti-Rabbit IgG antibody conjugated to horseradish peroxidase (1:5000; Bio-Rad), and Opti-4CN as optimized colorimetric substrate (Bio-Rad).

### Thermal stability assay

Protein melting curves to determine the thermal stability of PARPs were obtained using the fluorescent dye SYPRO Orange (Molecular Probes), as previous described^30,41^. Proteins (10 µg) were preincubated with 10X Sypro Orange (excitation at 490 nm, emission at 530 nm) in the presence of different buffers (100 mM), compounds (NAD^+^, ADP-ribose, nicotinamide, ammonium sulphate and hydroxyectoine) or inhibitors (3-aminobenzamidine). The assay, performed in 7500 RT-PCR equipment (Applied Biosystems), monitors Sypro Orange fluorescence while heating the samples from 5 to 98 °C. Each experiment was carried out in triplicate.

### *In silico* analysis

Protein sequences were obtained from NCBI non-redundant (NR) and UniProt databases using Clostridiales difficile CD 160 PARP protein sequence as a query. Incomplete sequences and duplicates were removed, rendering the sequences described in Supplementary Table S1. A Neighbour-Joining (NJ) tree with 1000 replicates was constructed using the MAFF server (https://mafft.cbrc.jp/alignment/server/). Domain architectures of retrieved sequences were obtained from the Pfam (http://www.sanger.ac.uk/Software/Pfam) and GenomeNet (http://www.genome.jp/tools/motif/) databases. Mutation correlation analysis was made with the retrieved bacterial PARP sequences, using Mistic (Mutual Information Server to Infer Coevolution) web server (http://mistic.leloir.org.ar)^27^. The x-ray structure of hPARP1 (PDB: 2DR6) was used as model for the *in silico* high-performance screening campaign on two complementary libraries of 50,000 compounds each (DIVERSet-EXP and DIVERSet-CL, ChemBridge) using LeadIT and SeeSAR (https://www.biosolveit.de/). Protein sequences were modelled with SwissModel^53^. Protein structure images and structural alignments were obtained with Chimera^54^. *Cdu1* gene phylogenetic three of *C. difficile* CD160 was constructed with MEGA 7.0^55^ using maximum likelihood method and the data provided by Prof. Marc Monot (Institute Pasteur, FR). PaLoc and CdTLoc representations of *C. difficile* strains were obtained from Patric web (https://www.patricbrc.org/).

## Electronic supplementary material

Supplementary Information

## References

[1] Ribet D, Cossart P (2010). Post-translational modifications in host cells during bacterial infection. FEBS letters.

[2] Palazzo L, Mikoc A, Ahel I (2017). ADP-ribosylation: new facets of an ancient modification. The FEBS journal.

[3] Hottiger MO (2015). Nuclear ADP-Ribosylation and Its Role in Chromatin Plasticity, Cell Differentiation, and Epigenetics. Annual review of biochemistry.

[4] Otto H (2005). In silico characterization of the family of PARP-like poly(ADP-ribosyl)transferases (pARTs). BMC genomics.

[5] Liu C, Yu X (2015). ADP-ribosyltransferases and poly ADP-ribosylation. Current protein & peptide science.

[6] Hottiger MO, Hassa PO, Luscher B, Schuler H, Koch-Nolte F (2010). Toward a unified nomenclature for mammalian ADP-ribosyltransferases. Trends in biochemical sciences.

[7] Steffen JD, Brody JR, Armen RS, Pascal JM (2013). Structural Implications for Selective Targeting of PARPs. Frontiers in oncology.

[8] Kleine H (2008). Substrate-assisted catalysis by PARP10 limits its activity to mono-ADP-ribosylation. Molecular cell.

[9] Crawford K, Bonfiglio JJ, Mikoc A, Matic I, Ahel I (2018). Specificity of reversible ADP-ribosylation and regulation of cellular processes. Critical reviews in biochemistry and molecular biology.

[10] Bock FJ, Chang P (2016). New directions in poly(ADP-ribose) polymerase biology. The FEBS journal.

[11] Barkauskaite E, Jankevicius G, Ladurner AG, Ahel I, Timinszky G (2013). The recognition and removal of cellular poly(ADP-ribose) signals. The FEBS journal.

[12] Pommier Y, O’Connor MJ, de Bono J (2016). Laying a trap to kill cancer cells: PARP inhibitors and their mechanisms of action. Science translational medicine.

[13] Lord CJ, Ashworth A (2017). PARP inhibitors: Synthetic lethality in the clinic. Science.

[14] Langelier MF, Planck JL, Roy S, Pascal JM (2012). Structural basis for DNA damage-dependent poly(ADP-ribosyl)ation by human PARP-1. Science.

[15] Karlberg T, Langelier MF, Pascal JM, Schuler H (2013). Structural biology of the writers, readers, and erasers in mono- and poly(ADP-ribose) mediated signaling. Molecular aspects of medicine.

[16] Perina D (2014). Distribution of protein poly(ADP-ribosyl)ation systems across all domains of life. DNA repair.

[17] Slade D (2011). The structure and catalytic mechanism of a poly(ADP-ribose) glycohydrolase. Nature.

[18] Patel CN, Koh DW, Jacobson MK, Oliveira MA (2005). Identification of three critical acidic residues of poly(ADP-ribose) glycohydrolase involved in catalysis: determining the PARG catalytic domain. The Biochemical journal.

[19] Kachrimanidou M, Malisiovas N (2011). Clostridium difficile infection: a comprehensive review. Critical reviews in microbiology.

[20] Lawson PA, Citron DM, Tyrrell KL, Finegold SM (2016). Reclassification of Clostridium difficile as *Clostridioides difficile* (Hall and O’Toole 1935) Prevot 1938. Anaerobe.

[21] Aktories K, Schwan C, Jank T (2017). Clostridium difficile Toxin Biology. Annual review of microbiology.

[22] Zhang S (2016). Cost of hospital management of Clostridium difficile infection in United States-a meta-analysis and modelling study. BMC infectious diseases.

[23] Just I (1995). Glucosylation of Rho proteins by Clostridium difficile toxin B. Nature.

[24] Monot M (2015). Clostridium difficile: New Insights into the Evolution of the Pathogenicity Locus. Scientific reports.

[25] Brouwer MS, Allan E, Mullany P, Roberts AP (2012). Draft genome sequence of the nontoxigenic Clostridium difficile strain CD37. Journal of bacteriology.

[26] Piepenbrink KH (2015). Structural and evolutionary analyses show unique stabilization strategies in the type IV pili of Clostridium difficile. Structure.

[27] Simonetti FL, Teppa E, Chernomoretz A, Nielsen M, Marino Buslje C (2013). MISTIC: Mutual information server to infer coevolution. Nucleic acids research.

[28] Gloor GB, Martin LC, Wahl LM, Dunn SD (2005). Mutual information in protein multiple sequence alignments reveals two classes of coevolving positions. Biochemistry.

[29] Petit D (2015). Integrative view ofalpha2,3-sialyltransferases (ST3Gal) molecular and functional evolution in deuterostomes: significance of lineage-specific losses. Molecular biology and evolution.

[30] Martinez-Monino AB (2017). Characterization and mutational analysis of a nicotinamide mononucleotide deamidase from Agrobacterium tumefaciens showing high thermal stability and catalytic efficiency. PloS one.

[31] Langelier MF, Riccio AA, Pascal JM (2014). PARP-2 and PARP-3 are selectively activated by 5′ phosphorylated DNA breaks through an allosteric regulatory mechanism shared with PARP-1. Nucleic acids research.

[32] Citarelli M, Teotia S, Lamb RS (2010). Evolutionary history of the poly(ADP-ribose) polymerase gene family in eukaryotes. BMC evolutionary biology.

[33] Dingle KE (2014). Evolutionary history of the Clostridium difficile pathogenicity locus. Genome biology and evolution.

[34] Tan ES, Krukenberg KA, Mitchison TJ (2012). Large-scale preparation and characterization of poly(ADP-ribose) and defined length polymers. Analytical biochemistry.

[35] Nottbohm AC, Dothager RS, Putt KS, Hoyt MT, Hergenrother PJ (2007). A colorimetric substrate for poly(ADP-ribose) polymerase-1, VPARP, and tankyrase-1. Angew Chem Int Ed Engl.

[36] Langelier MF, Ruhl DD, Planck JL, Kraus WL, Pascal JM (2010). The Zn3 domain of human poly(ADP-ribose) polymerase-1 (PARP-1) functions in both DNA-dependent poly(ADP-ribose) synthesis activity and chromatin compaction. The Journal of biological chemistry.

[37] Altmeyer M, Messner S, Hassa PO, Fey M, Hottiger MO (2009). Molecular mechanism of poly(ADP-ribosyl)ation by PARP1 and identification of lysine residues as ADP-ribose acceptor sites. Nucleic acids research.

[38] Putt KS, Hergenrother PJ (2004). An enzymatic assay for poly(ADP-ribose) polymerase-1 (PARP-1) via the chemical quantitation of NAD(+): application to the high-throughput screening of small molecules as potential inhibitors. Analytical biochemistry.

[39] Zhu Z, Jin J, Xue N, Song X, Chen X (2014). Development and validation of high-throughput screening assays for poly(ADP-ribose) polymerase-2 inhibitors. Analytical biochemistry.

[40] Wahlberg E (2012). Family-wide chemical profiling and structural analysis of PARP and tankyrase inhibitors. Nature biotechnology.

[41] Zapata-Perez, R. *et al*. Structural and functional analysis of Oceanobacillus iheyensis macrodomain reveals a network of waters involved in substrate binding and catalysis. *Open biology* **7**, 10.1098/rsob.160327 (2017). 10.1098/rsob.160327 PMC5413906 28446708

[42] Graeff R, Lee HC (2002). A novel cycling assay for cellular cADP-ribose with nanomolar sensitivity. The Biochemical journal.

[43] Graeff R, Lee HC (2002). A novel cycling assay for nicotinic acid-adenine dinucleotide phosphate with nanomolar sensitivity. The Biochemical journal.

[44] Zembower DE, Singh J, Mishra RK (2010). Small Moleculle Modulators of Cytokine Activity. US.

[45] Mahasenan KV, Li C (2012). Novel inhibitor discovery through virtual screening against multiple protein conformations generated via ligand-directed modeling: a maternal embryonic leucine zipper kinase example. Journal of chemical information and modeling.

[46] Antolin AA (2013). Exploring the effect of PARP-1 flexibility in docking studies. Journal of molecular graphics & modelling.

[47] Jagtap PG (2004). The discovery and synthesis of novel adenosine substituted 2,3-dihydro-1H-isoindol-1-ones: potent inhibitors of poly(ADP-ribose) polymerase-1 (PARP-1). Bioorganic & medicinal chemistry letters.

[48] Haikarainen T, Narwal M, Joensuu P, Lehtio L (2014). Evaluation and Structural Basis for the Inhibition of Tankyrases by PARP Inhibitors. ACS medicinal chemistry letters.

[49] Rack JG, Perina D, Ahel I (2016). Macrodomains: Structure, Function, Evolution, and Catalytic Activities. Annual review of biochemistry.

[50] Jankevicius G, Ariza A, Ahel M, Ahel I (2016). The Toxin-Antitoxin System DarTG Catalyzes Reversible ADP-Ribosylation of DNA. Molecular cell.

[51] Sanchez-Carron G (2011). Molecular characterization of a novel N-acetylneuraminate lyase from Lactobacillus plantarum WCFS1. Applied and environmental microbiology.

[52] Langelier MF, Planck JL, Servent KM, Pascal JM (2011). Purification of human PARP-1 and PARP-1 domains from Escherichia coli for structural and biochemical analysis. Methods Mol Biol.

[53] Biasini M (2014). SWISS-MODEL: modelling protein tertiary and quaternary structure using evolutionary information. Nucleic acids research.

[54] Pettersen EF (2004). UCSF Chimera–a visualization system for exploratory research and analysis. Journal of computational chemistry.

[55] Kumar S, Stecher G, Tamura K (2016). MEGA7: Molecular Evolutionary Genetics Analysis Version 7.0 for Bigger Datasets. Molecular biology and evolution.

[56] Ihnen M (2013). Therapeutic potential of the poly(ADP-ribose) polymerase inhibitor rucaparib for the treatment of sporadic human ovarian cancer. Molecular cancer therapeutics.

[57] Donawho CK (2007). ABT-888, an orally active poly(ADP-ribose) polymerase inhibitor that potentiates DNA-damaging agents in preclinical tumor models. Clinical cancer research: an official journal of the American Association for Cancer Research.

[58] Garcia Soriano F (2001). Diabetic endothelial dysfunction: the role of poly(ADP-ribose) polymerase activation. Nature medicine.

[59] Eliasson MJ (1997). Poly(ADP-ribose) polymerase gene disruption renders mice resistant to cerebral ischemia. Nature medicine.

[60] Banasik M, Komura H, Shimoyama M, Ueda K (1992). Specific inhibitors of poly(ADP-ribose) synthetase and mono(ADP-ribosyl)transferase. The Journal of biological chemistry.

[61] Chiarugi A (2003). Novel isoquinolinone-derived inhibitors of poly(ADP-ribose) polymerase-1: pharmacological characterization and neuroprotective effects in an *in vitro* model of cerebral ischemia. The Journal of pharmacology and experimental therapeutics.

[62] Huang SM (2009). Tankyrase inhibition stabilizes axin and antagonizes Wnt signalling. Nature.

