# Supplementary material for: Comparative inhibitory profile and distribution of bacterial PARPs, using Clostridioides difficile CD160 PARP as a model
Source: Sci Rep. 2018 May 23;8:8056. doi: 10.1038/s41598-018-26450-0 (PMC5966428; doi:10.1038/s41598-018-26450-0)
Supplement: Supplementary file 1 — Supplementary Information [file 41598_2018_26450_MOESM1_ESM.pdf]

Supplementary Information

Comparative inhibitory profile and distribution of bacterial PARPs, using *Clostridioides difficile* CD160 PARP as a model.

Authors:

Antonio Ginés García-Saura, Rubén Zapata-Pérez, José Francisco Hidalgo, Álvaro Sánchez-Ferrer*

Affiliations:

Department of Biochemistry and Molecular Biology-A, Faculty of Biology, Regional Campus of International Excellence "Campus Mare Nostrum", University of Murcia, Campus Espinardo, E-30100 Murcia, Spain.

Murcia Biomedical Research Institute (IMIB-Arrixaca), 30120 Murcia, Spain

UniProt Code	Phylum	Order	Species
A0A0J8TZQ7	Actinobacteria	Corynebacteriales	*Mycobacterium conceptionense*
A0A0R3ELX8	Actinobacteria	Corynebacteriales	*Mycobacterium sp.* H092
A0A0S9KD90	Actinobacteria	Micrococcales	*Leifsonia sp.* Leaf264
A0A0U1BS85	Actinobacteria	Corynebacteriales	*Mycobacterium abscessus*
A0A100WBB7	Actinobacteria	Corynebacteriales	*Mycobacterium canariasense*
A0A193K1I5	Actinobacteria	Corynebacteriales	*Mycobacterium abscessus*
A0A1H4UY96	Actinobacteria	Corynebacteriales	*Tsukamurella tyrosinosolvens*
A0A1M8LI26	Actinobacteria	Corynebacteriales	*Mycobacterium abscessus* subsp. *abscessus*
A0A1M9H3A7	Actinobacteria	Corynebacteriales	*Mycobacterium abscessus* subsp. *abscessus*
A0A1N0R071	Actinobacteria	Corynebacteriales	*Mycobacterium abscessus* subsp. *bolletii*
A0A1N3ZHF0	Actinobacteria	Corynebacteriales	*Mycobacterium abscessus* subsp. *abscessus*
A0A1Q9W8C2	Actinobacteria	Corynebacteriales	*Mycobacterium sp.* 27335
A0A1R4KIH9	Actinobacteria	Micrococcales	*Microbacterium esteraromaticum*
A0A1S1K222	Actinobacteria	Corynebacteriales	*Mycobacterium sp.* 24999
A0A1U1BJN9	Actinobacteria	Corynebacteriales	*Mycobacterium abscessus* subsp. *massiliense*
A0A1U6E4C5	Actinobacteria	Corynebacteriales	*Mycobacterium abscessus* subsp. *massiliense*
A0A1X0ITS8	Actinobacteria	Corynebacteriales	*Mycobacterium saopaulense*
A0A1Y0C5Q4	Actinobacteria	Corynebacteriales	*Mycobacterium dioxanotrophicus*
T5KJ81	Actinobacteria	Micrococcales	*Microbacterium maritypicum* MF109
X8DPT9	Actinobacteria	Corynebacteriales	*Mycobacterium abscessus* subsp. *bolletii* 1513
A0A1Q3GNY7	Bacteroidetes	Cytophagales	*marine bacterium* AO1-C
A0A1Q3GQ85	Bacteroidetes	Cytophagales	*marine bacterium* AO1-C
A0A1Q3WEP4	Bacteroidetes	Cytophagales	*Spirosoma sp.* 48-14
A0A1S2VEW1	Bacteroidetes	Cytophagales	*Arsenicibacter rosenii*
A0A1V5GYX3	Bacteroidetes		*Bacteroidetes bacterium* ADurb.BinA174
A0A1W6E457	Bacteroidetes	Cytophagales	*Fibrella sp.* ES10-3-2-2
A1ZG37	Bacteroidetes	Cytophagales	*Microscilla marina* ATCC 23134
A2A044	Bacteroidetes	Cytophagales	*Microscilla marina* ATCC 23134
D2QNL1	Bacteroidetes	Cytophagales	*Spirosoma linguale* (strain ATCC 33905 / DSM 74 / LMG 10896)
I0K3M8	Bacteroidetes	Cytophagales	*Fibrella aestuarina* BUZ 2
I2GN74	Bacteroidetes	Cytophagales	*Fibrisoma limi* BUZ 3
I4AHF7	Bacteroidetes	Cytophagales	*Bernardetia litoralis* (strain ATCC 23117 / DSM 6794 / NBRC 15988 / NCIMB 1366 / Sio-4)
A0A0P6Y792	Chloroflexi	Herpetosiphonales	*Herpetosiphon geysericola*
A9B244	Chloroflexi	Herpetosiphonales	*Herpetosiphon aurantiacus* (strain ATCC 23779 / DSM 785)
A0A140K273	Cyanobacteria	Pleurocapsales	*Stanieria sp.* NIES-3757
K9XWE7	Cyanobacteria	Pleurocapsales	*Stanieria cyanosphaera* (strain ATCC 29371 / PCC 7437)
A0A0B5AXL3	Firmicutes	Bacillales	*Jeotgalibacillus malaysiensis*
A0A0D0SCB8	Firmicutes	Clostridiales	*Lachnospiraceae bacterium* TWA4
A0A0F6J8Z7	Firmicutes	Bacillales	*Bacillus thuringiensis* T01-328
A0A150D4X5	Firmicutes	Bacillales	*Bacillus cereus*
A0A173XPD6	Firmicutes	Clostridiales	*[Eubacterium] hallii*

A0A174EUZ4	Firmicutes	Clostridiales	*Dorea longicatena*
A0A174EXW8	Firmicutes	Clostridiales	*Dorea longicatena*
A0A174FXB1	Firmicutes	Clostridiales	*Blautia obeum*
A0A174NRB4	Firmicutes	Clostridiales	*Anaerostipes hadrus*
A0A174VXZ8	Firmicutes	Clostridiales	*Blautia obeum*
A0A1C5NAB2	Firmicutes	Clostridiales	*uncultured Clostridium sp.*
A0A1V5KBH0	Firmicutes		*Firmicutes bacterium* ADurb.Bin506
A0A1Y4B883	Firmicutes	Clostridiales	*Eubacterium sp.* An3
A0A1Y4HI40	Firmicutes	Clostridiales	*Anaerofilum sp.* An201
A0A1Y6ATX8	Firmicutes	Bacillales	*Bacillus cereus*
D4JQ95	Firmicutes	Clostridiales	*[Eubacterium] rectale* M104/1
E0S444	Firmicutes	Clostridiales	*Butyrivibrio proteoclasticus* (strain ATCC 51982 / DSM 14932 / B316)
E3EKH1	Firmicutes	Bacillales	*Paenibacillus polymyxa* (strain SC2)
R9MCJ0	Firmicutes	Clostridiales	*Lachnospiraceae bacterium* 3-2
T3DQ72	Firmicutes	Clostridiales	*Clostridioides difficile CD160*
A0A0C1ZPK8	Proteobacteria	Myxococcales	*Enhygromyxa salina*
A0A0F6W421	Proteobacteria	Myxococcales	*Sandaracinus amylolyticus*
A0A0H4JRL6	Proteobacteria	Vibrionales	*Vibrio cholerae*
A0A0L7YS65	Proteobacteria	Vibrionales	*Vibrio parahaemolyticus*
A0A0N1JKQ8	Proteobacteria	Pseudomonadales	*Pseudomonas amygdali pv. lachrymans*
A0A0Q4NHG1	Proteobacteria	Pseudomonadales	*Pseudomonas sp.* Leaf58
A0A109E1S6	Proteobacteria	Burkholderiales	*Burkholderia cenocepacia*
A0A120DEW5	Proteobacteria	Burkholderiales	*Variovorax sp.* WDL1
A0A1F9FGL8	Proteobacteria		*Deltaproteobacteria bacterium* RBG_16_71_12
A0A1F9KE67	Proteobacteria		*Deltaproteobacteria bacterium* RIFOXYA12_FULL_58_15
A0A1F9L7H8	Proteobacteria		*Deltaproteobacteria bacterium* RIFOXYA12_FULL_58_15
A0A1F9MZ55	Proteobacteria		*Deltaproteobacteria bacterium* RIFOXYB12_FULL_58_9
A0A1F9N6B2	Proteobacteria		*Deltaproteobacteria bacterium* RIFOXYB12_FULL_58_9
A0A1W6MEK5	Proteobacteria	Vibrionales	*Vibrio vulnificus*
A6FZA8	Proteobacteria	Myxococcales	*Plesiocystis pacifica* SIR-1
S7HV61	Proteobacteria	Vibrionales	*Vibrio fluvialis* PG41

Supplementary Table S1. Selected bacterial PARPs found in NCBI non-redundant (NR) and UniProt databases.

Condition	hPARP1	HaPARP	CdPARP
	*T*m	*T*m	*T*m
MilliQ® water	45,88±0,11	39,75±0,15	40,27±0,09
pH 6.0	45,28±0,10	35,52±0,11	39,55±0,15
pH 7.0	46,33±0,13	39,23±0,08	44,3±0,11
pH 7.5	46,80±0,17	39,61±0,07	44,97±0,05
pH 8.0	46,57±0,09	40,26±0,13	41,15±0,04
pH 8.5	46,09±0,10	40,35±0,10	40,74±0,11
pH 9.0	45,58±0,12	40,04±0,05	40,21±0,12
pH 10.0	44,27±0,08	38,25±0,14	39,61±0,05
Ammonium sulphate (1 M)	48,66±0,09	41,97±0,12	46,4±0,03
Hydroxyectoine (1 M)	50,93±0,11	44,73±0,11	43,21±0,11
NAD^+ (1 mM)	44,97±0,06	39,57±0,06	37,92±0,12
ADP-ribose (1 mM)	45,28±0,11	39,18±0,09	39,51±0,13
Nicotinamide (1 mM)	48,37±0,09	43,26±0,11	41,91±0,08
3-Aminobenzamidine (1 mM)	48,94±0,12	46,91±0,10	41,96±0,06

Supplementary Table S2. Melting temperature (*T*m) values of hPARP1, HaPARP and CdPARP under different conditions.

	hPARP1	CdPARP	HaPARP
**Compound**	**Inhibition (%)**		
Rucaparib	100.0	100.0	100.0
ABT-888	100.0	47.5±5.1	37.1±3.1
DPQ	100.0	53.7±2.4	21.1±2.6
EB-47	100.0	100.0	100.0
PJ-34	100.0	49.8±5.6	38.0±2.9
4-amino 1.8-naphthalimide	98.9±0.6	9.2±1.2	12.7±1.8
TIQ-A	94.64±0.3	62.1±4.3	80.2±5.8
XAV-939	94.32±5.2	36.3±2.1	45.7±3.7
7650155	91.00±0.9	13.5±1.2	3.1±0.6
7651361	89.91±3.4	11.2±1.3	2.7±0.4
9019116	84.14±5.4	18.0±3.0	10.7±0.8
9064221	79.44±0.9	4.6±1.1	11.0±1.1
9155111	77.43±7.8	9.8±0.2	9.5±1.0
59544699	74.69±0.8	5.13±0.9	32.4±2.5
7650649	70.03±0.3	1.0±0.1	9.6±0.5
53741273	68.7±5.7	0.0	22.8±0.6
7670490	67.29±9.3	24.2±4.2	3.2±0.9
7655698	66.83±0.2	2.3±0.5	0.3±0.1
7669941	65.77±2.1	5.7±0.4	0.0
7642078	65.40±0.7	10.2±0.9	6.4±0.5
7660328	62.00±0.5	11.2±1.7	0.0
7912202	60.58±0.9	20.7±3.4	5.7±0.9
9075009	56.81±2.8	0.0	3.6±0.5
7803039	48.01±6.6	19.5±2.3	3.1±0.7
27904679	45.76±2.6	10.1±0.8	16.4±2.3
9128661	36.57±8.3	0.0	15.3±1.9
9017662	32.97±0.2	6.8±0.7	7.0±0.7
9138821	31.63±0.8	2.3±0.7	9.2±1.1
9135871	30.39±2.4	0.0	18.2±0.4
9133307	30.02±9.3	1.2±1.1	8.9±0.2
9150050	29.13±0.7	7.3±1.6	1.7±0.6
9077168	29.06±1.9	21.4±3.7	5.7±0.3
14009467	20.05±2.3	0.2±0.1	18.5±1.7
12532172	17.62±4.5	11.7±1.9	7.2±0.7
9131621	17.16±5.6	3.9±0.6	6.1±0.8
UPF1069	13.20±3.4	15.2±2.6	13.3±1.2
13564290	12.90±6.1	0.0	18.1±2.1
20219443	8.51±2.1	9.8±1.7	22.2±2.8
19421550	5.99±0.2	4.7±2.1	9.4±0.7
9152982	0.0	2.2±0.1	0.0
18588770	0.0	2.7±0.8	7.0±0.9
11386820	0.0	0.1	0.0
29348813	0.0	7.0±2.1	12.7±2.3
31066495	0.0	6.5±1.2	13.9±0.8

Supplementary Table S3. Effect of selected compounds on PARP activity. These compounds were assayed as described in Materials and Methods section.

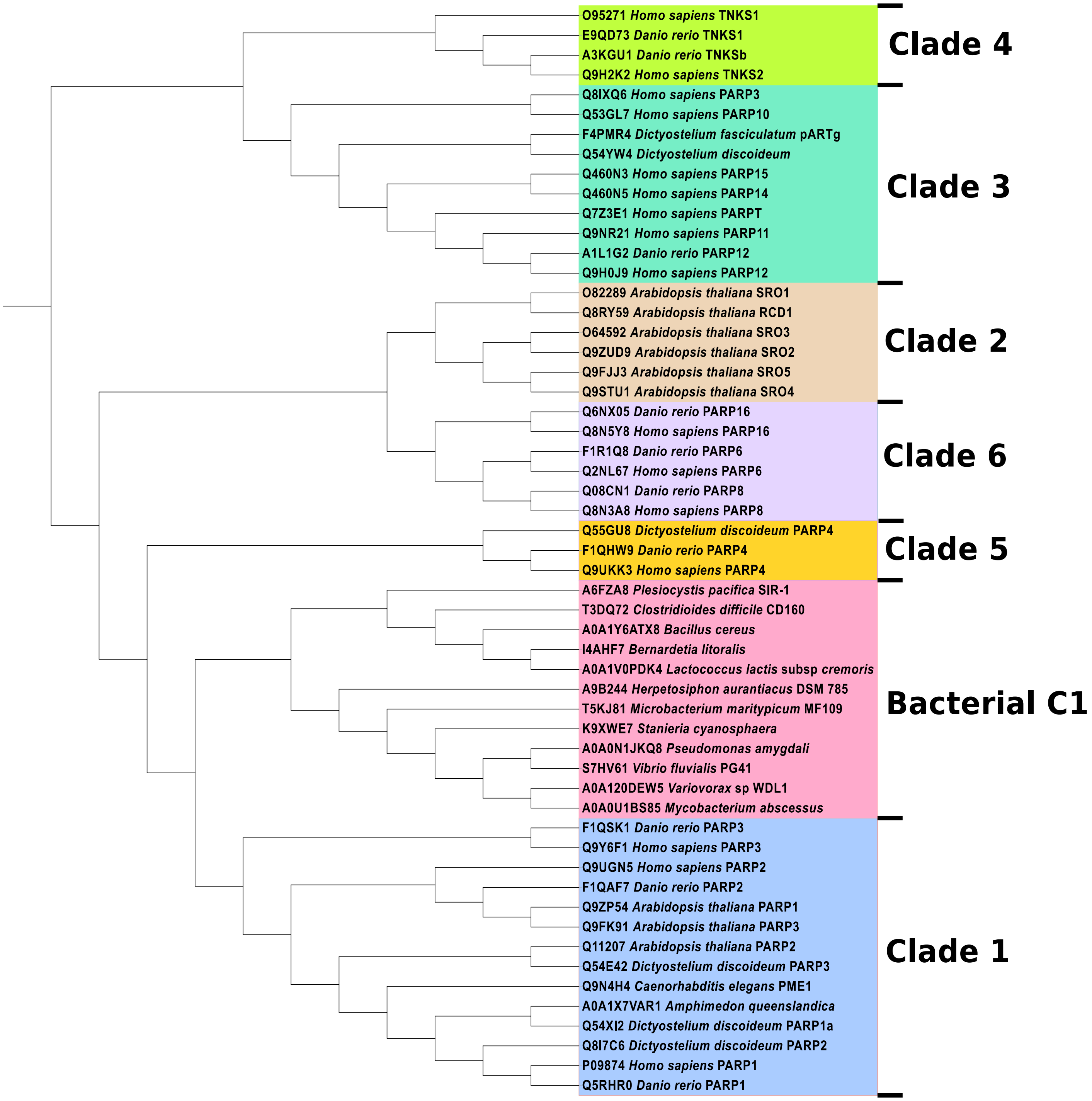

Supplementary Fig. SI. Phylogenetic distribution of the catalytic PARP domains from representative eukaryotic and bacterial PARPs. Neighbour-Joining (NJ) tree with 1000 replicates obtained using the MAFF server.

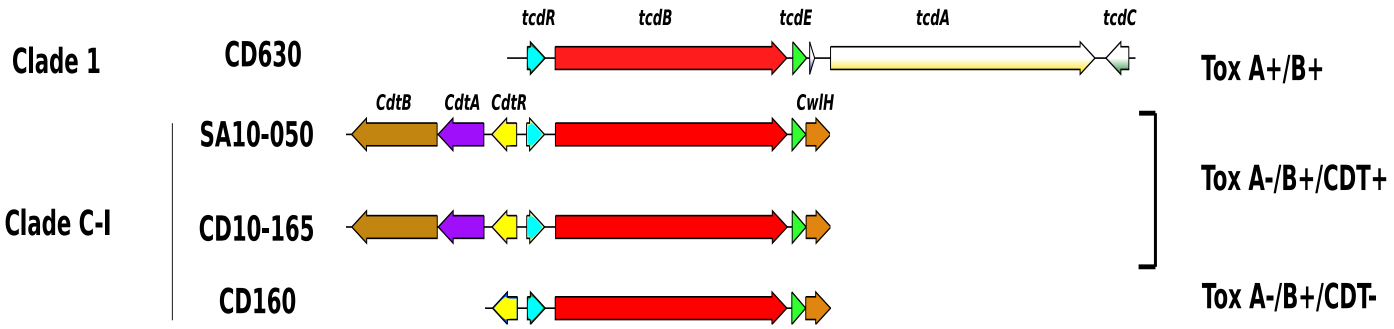

Supplementary Fig. S2. PaLoc organization of the selected Clade 1 and C-I *C. difficile* strains. CD630 (type strain), SA10-50 and CD10-165 are toxigenic representatives from clades C1 and C-I respectively.

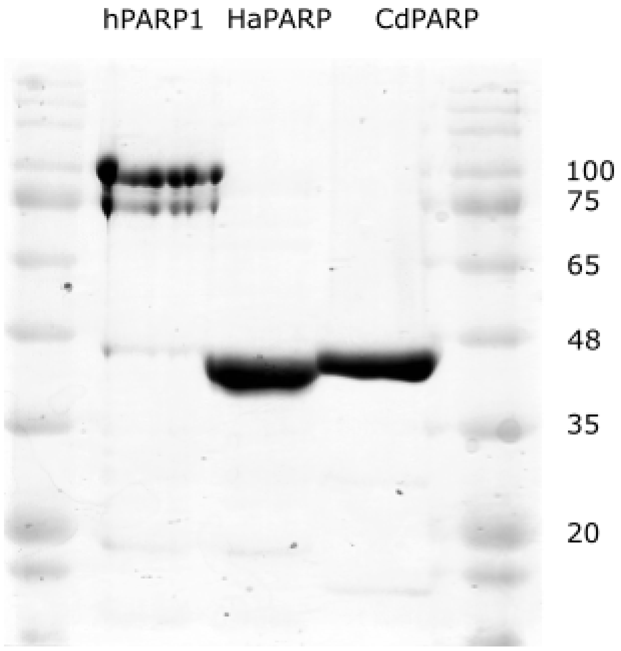

Supplementary Fig. S3. SDS-PAGE (10%) of purified hPARP1, HaPARP and CdPARP**.** Molecular mass standards (kDa) are indicated in the right margin.

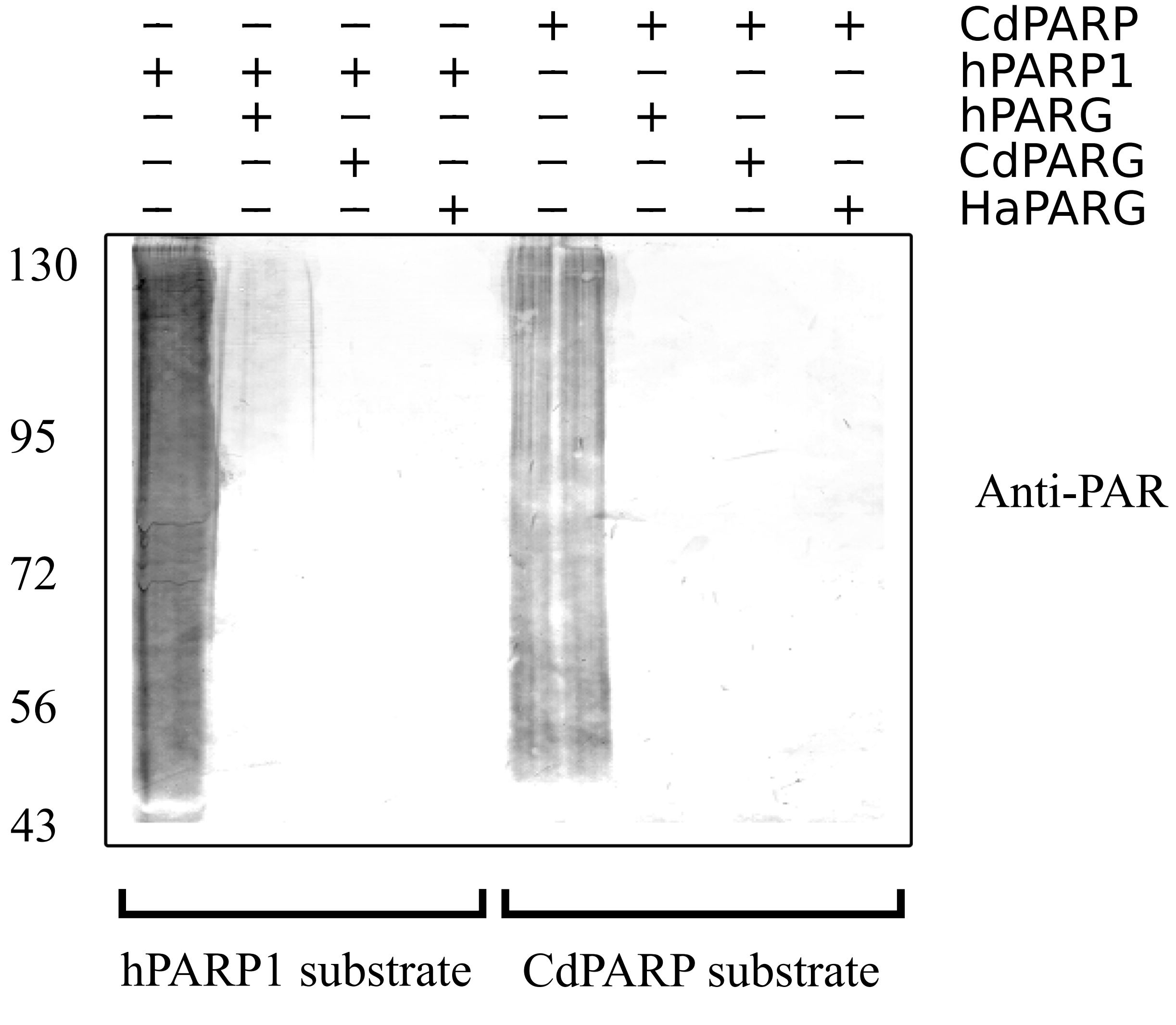

Supplementary Fig. S4. PAR glycohydrolase activity of hPARPG, CdPARG and HaPARG. Western blot analysis was carried out with anti-PAR antibodies as described in Materials and Methods section.

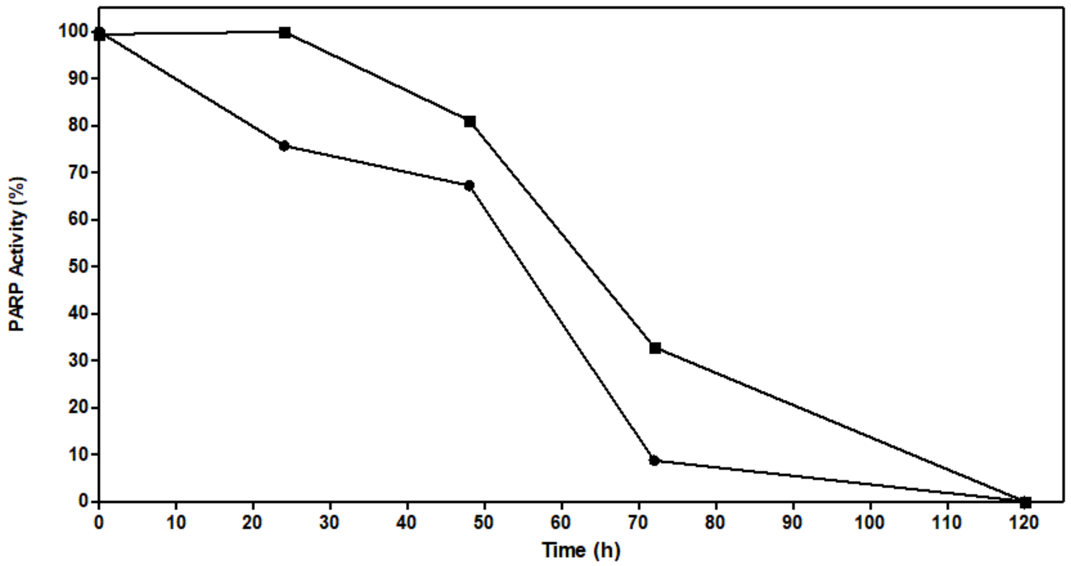

Supplementary Fig. S5. Effect of protein stabilizers on hPARP1 stability. (■) Hydroxyectoine (200 mM). (●) Glycerol (10%).

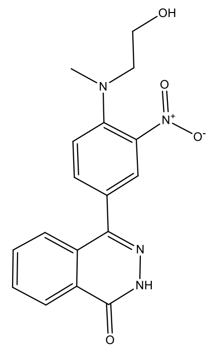

7655698
4-{4-[(2-hydroxyethyl)(methyl)amino]-3-nitrophenyl}-1(2H)-phthalazinone

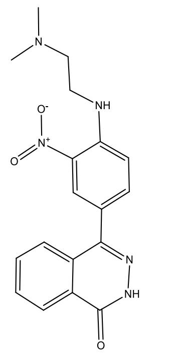

7650649
4-(4-{[2-(dimethylamino)ethyl]amino}-3-nitrophenyl)-1(2H)-phthalazinone

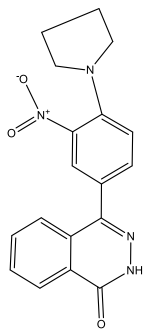

7651361
4-[3-nitro-4-(1-pyrrolidinyl)phenyl]-1(2H)-phthalazinone

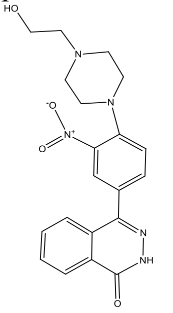

7642078
4-{4-[4-(2-hydroxyethyl)-1-piperazinyl]-3-nitrophenyl}-1(2H)-phthalazinone

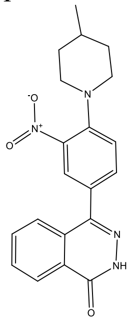

7669941
4-[4-(4-methyl-1-piperidinyl)-3-nitrophenyl]-1(2H)-phthalazinone

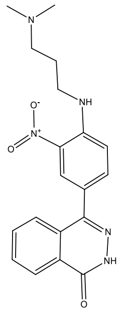

7660328
4-(4-{[3-(dimethylamino)propyl]amino}-3-nitrophenyl)-1(2H)-phthalazinone

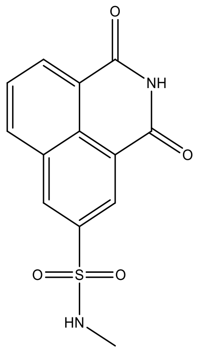

7670490
N-methyl-1,3-dioxo-2,3-dihydro-1H-benzo[de]isoquinoline-5-sulfonamide

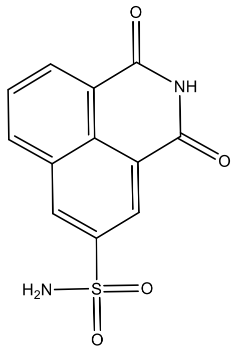

7650155
1,3-dioxo-2,3-dihydro-1H-benzo[de]isoquinoline-5-sulfonamide

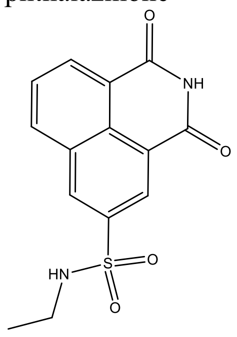

9019116
N-ethyl-1,3-dioxo-2,3-dihydro-1H-benzo[de]isoquinoline-5-sulfonamide

Supplementary Fig. S6. Chemical structures of the most active new compounds towards hPARP1 described in Table 1.
